# Accumulation of nanoparticles in “jellyfish” mucus: a bio-inspired route to decontamination of nano-waste

**DOI:** 10.1038/srep11387

**Published:** 2015-06-22

**Authors:** Amit Patwa, Alain Thiéry, Fabien Lombard, Martin K.S. Lilley, Claire Boisset, Jean-François Bramard, Jean-Yves Bottero, Philippe Barthélémy

**Affiliations:** 1 INSERM U869, Bordeaux, F-33076, France; 2 Université de Bordeaux, Bordeaux, F-33076, France; 3 IMBE UMR CNRS 7263, Aix-Marseille Université, Aix en Provence F-13545, France; 4 Sorbonne Universités, UPMC Univ Paris 06, UMR 7093, LOV, Observatoire océanologique, 06230, Villefranche sur mer, France; 5 CERMAV, CNRS UPR 5301, Grenoble, F-38041, France; 6 Cambulle, 171C, av. de la Mounine, 13320 Bouc Bel-Air; 7 CEREGE Europole de l’Arbois BP 80, Aix en Provence F-13545, France

## Abstract

The economic and societal impacts of nano-materials are enormous. However, releasing such materials in the environment could be detrimental to human health and the ecological biosphere. Here we demonstrate that gold and quantum dots nanoparticles bio-accumulate into mucus materials coming from natural species such as jellyfish. One strategy that emerges from this finding would be to take advantage of these trapping properties to remove nanoparticles from contaminated water.

In parallel to the technological benefits expected from the impressive development of nanotechnologies, the arrival on the market of nano-products raises crucial issues dealing with human/environmental risk assessment^1^ and potential associated contaminations^2^. Nanoscale materials are used in a variety of different areas such as electronic^3^, biomedical^4^,^5^,^6^, nanophotonics^7^, cosmetic, energy^8^, and engineering^9^. In line with increasing production and use of nanoparticles over the next years, it is anticipated that these particles will be released into the environment during the production, transport or disposal processes. Indeed, production and manipulation of nanomaterials must be safe for everyone, meaning that companies, laboratories and/or factories structures must integrate the decontamination dimension in their activities. Addressing these decontamination problems calls out for research to be conducted to identify robust new methods of decontaminating aqueous nano-wastes at lower cost and with less energy, while at the same time minimizing the impact on the environment. Surprisingly, while some bioremediations of domestic waters exists during lagooning procedure of sewage treatments and despite the strong demand of producers and users of nanoparticles product, only a few types of decontamination devices have been seriously considered^10^. Recently, we discovered that hydrogel-based materials containing supramolecular systems could be used for the decontamination of aqueous samples containing nanoparticles (NPs)^11^. Though references are available on treatment of water using bacteria and fungus^12^,^13^ through bio-accumulation, bio-flocculation and bioremediation mechanisms^14^,^15^, from our knowledge no literature is available on biomaterials capable to quickly and efficiently remove NPs from wastewater originating from nanoparticle factories. Thus, since it was previously reported that nanoparticles were uptaken by several organisms^16^,^17^,^18^,^19^,^20^,^21^, it was conceived that decontamination alternatives might be discovered from biological systems capable of bio-accumulation of NPs. In this work, we hypothesized that mucus coming from natural resources could be used as a biomaterial to capture NPs. Here we demonstrate that gold and quantum dot nanoparticles bio-accumulate into mucus materials coming from natural species such as “jellyfish” (Fig. 1). We refer to “jellyfish” in the commonly used *sensus lato*^22^, which include jellyfish *sensus stricto* (Cnidaria) but also comb jellyfish (Ctenophora).

Jellyfish have been the subject of several applications in biotechnology, such as glowing tissues, green fluorescent proteins that have become a massively useful tool in biological science and medicine (Shimomura, Chalfie & Tsien obtaining the Nobel Peace Prize in 2008)^23^,^24^,^25^,^26^, or as model for biomimetic propulsion^27^. However, to our knowledge no data are available on the use of excreted mucus and its properties. In this contribution we investigated the recycling potential of jellyfish, as bycatch or cultured specifically for industry, to decontaminate wastewaters. In this context, we studied the bio-accumulation and the potential contamination of jellyfish-based materials with NPs dissolved in marine waters. Three species, the Moon Jellyfish *Aurelia aurita*, the Mauve Stinger *Pelagia noctiluca*, and the invasive Ctenophore warty comb jelly *Mnemiopsis leidyi* were investigated.

## Results

**Figure 2:**
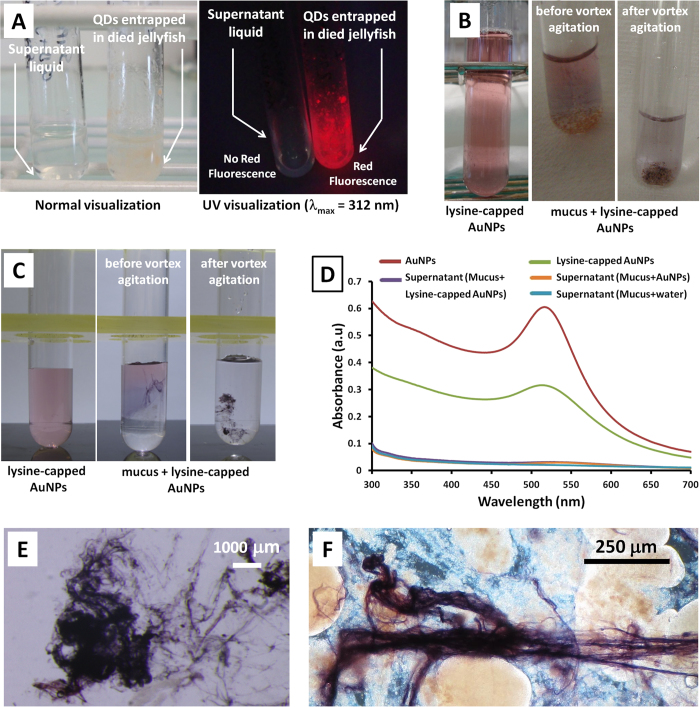
(**A**) Warty comb jelly (*Mnemiopsis leidyi*) with quantum dots (QDs) (**B**) Reproduction mucus (from *Pelagia noctiluca*) with lysine-capped AuNPs (**C**) Mucus (from *Aurelia aurita*) with lysine-capped AuNPs (D) UV-visible absorption spectra for reproduction mucus (*Pelagia noctiluca*) with lysine-capped AuNPs (10^−4^ M) (**E**) Optical microscopy image of mucus (*Aurelia aurita*) with lysine-capped AuNPs (10^−4^ M) (**F**) Optical microscopy image for reproduction mucus (*Pelagia noctiluca*) with lysine-capped AuNPs.

Initially, live *Mnemiopsis leidyi* were incubated in the presence of quantum dot nanoparticles (lipid encapsulated QDs, size (diameter) = 15 nm)^28^. Immediately after mixing, the glass tubes containing these mixtures (QDs) were examined under UV light (λ_max_ = 312 nm). The reaction mixture was fluorescent (red) except for the warty comb jelly portion, which appeared as black spots without any red fluorescence (Figure S1). This meant that the QDs were not able to penetrate into the tissue of the comb jelly. After settling for 48 h, during which time the jellyfish had died and disintegrated into small fluffy aggregates, the whole solution was observed to fluoresce under UV light. After 72 h the solution became transparent with some of fluffy particles precipitated to the bottom and others stuck to the sides of the glass tube (Figure S2). Examination of the transparent supernatant under UV light showed no red fluorescence, confirming that almost all of the QDs had been absorbed by the dead jellyfish (Fig. 2). It is notable that the QDs were only precipitated from the solution when the comb jelly died. We considered the potential role of mesoglea among animal groups in trapping particles because gel material may contribute to NP-Gel interactions. However, this material was unable to trap efficiently the NPs, indicating that other biomolecules secreted by the gelatinous animal were responsible for the capture of the NPs.

Unlike *M. leidyi,* the other jellyfish species studied were found to secrete a neutrally-buoyant mucus frequently without dying in the process. We also observed mucus produced by *P. noctiluca* and *A. aurita* during reproduction^29^, when stressed (e.g. handling or disturbance, see SI, Movie S1), and during digestion (to precipitate rejected particles from the water column). Both species also produced mucus when dying. All of these mucus have the ability to bind together in the same way as observed in *M. leidyi,* but were not always effective in trapping nanoparticles.

In the second series of experiments, some of these fresh jellyfish mucus were tested with gold nanoparticles (AuNPs and lysine capped AuNPs (size (diameter) = 6.5 ± 7 nm), Fig. 2B)^30^. The entrapment of AuNPs was visually assessed through both the disappearance of the typically ruby red color of AuNPs from the supernatant and through the obvious dark/pink-purple AuNPs aggregations inside the mucus (Fig. 2C). The stress mucus of *A. aurita* and reproduction mucus of *P. noctiluca* were particularly effective in capturing AuNPs. In order to evaluate the trapping efficiency of these mucuses, UV-visible absorption experiments were performed. In a typical experiment, reproduction mucus (500 μL) secreted by *Pelagia noctiluca* was incubated in the presence of lysine-capped AuNPs or uncapped AuNPs (1.0 mL). Figure 2D shows the absorbance of the initial NPs samples (red and green curves) and the supernatants after adding the mucus (orange and purple curves). Importantly, the absorbance of the supernatants in the range 500–550 nm of both lysine capped and uncapped AuNPs experiments were equal to the control experiment (NP free samples, blue curve), demonstrating that mucus captured quantitatively the AuNPs. Optical microscopy images of mucus secreted by *Aurelia aurita* and *Pelagia noctiluca* after incubation in the presence of lysine-capped AuNPs are shown in Fig. 2E,F, respectively. Interestingly, the purple color observed in these samples indicate that the NPs bioaccumulate in the mucus fibers.

**Figure 3:**
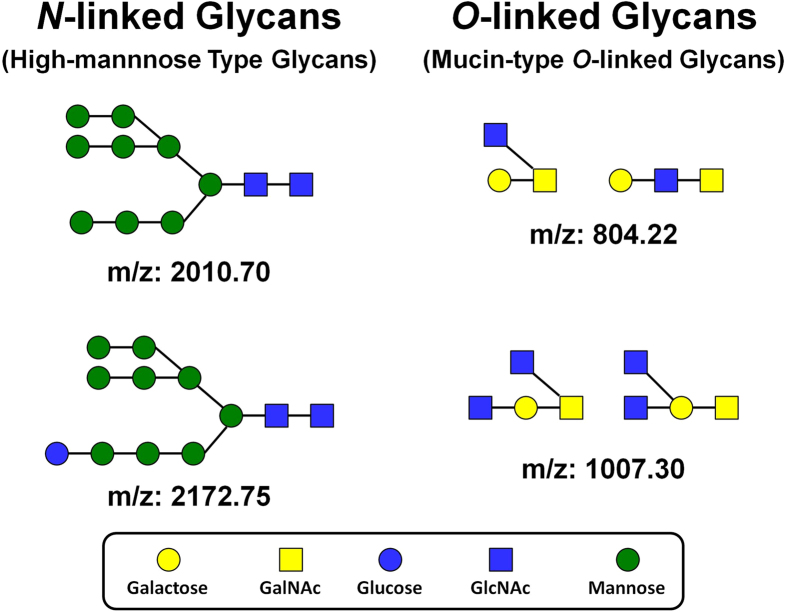
Examples of the chemical structures of High-mannose type *N*-linked glycans and mucin-type *O*-linked glycans found in the mucus of *Pelagia noctiluca*.

To identify the chemical nature of the material featuring the trapping properties, a series of analyses were performed. Large amounts of glycoproteins, namely mucin are found in almost every organ of jellyfish^31^. Jellyfish are known to secret mucus in different situations, including stress to clean their surface and to defend themselves against attacks from predators^32^. Here, the monosaccharide composition of reproduction mucus (*Pelagia noctiluca*) was determined in triplicate by Gas Chromatography. The results reveal the presence of Arabinose, Glucose, Mannose, GalNAc, GlcNAc with the following molar ratios: 1, 0.33, 0.42, 0.65, 0.56, respectively (See Table S4). Note that protein concentration of the mucus was estimated to be 0.6 mg/mL, indicating that glycoproteins are present in the mucus. The two major forms of protein glycosylation are *N*- and *O*-glycosylation, hence a second series of analyses were used to determine the chemical structures of the *O*-linked and *N*-linked glycans. In a typical experiment, the mucus was analyzed in duplicate to quantify both *N*-linked and *O*-linked glycans using a general protocol for the isolation and analysis by MALDI-TOF mass spectrometry of carbohydrates, which is based on the methods previously reported by Nishimura *et al.*, Miura *et al.* and Furukawa *et al.*^33^,^34^,^35^. In the case of *N*-linked glycans, this analysis detects both *N*-linked glycans and oligosaccharides with free reducing ends (free oligosaccharides; FOS). Three types of oligosaccharides were detected in the mucus sample (*Pelagia noctiluca):* a) High-mannose type *N*-linked glycans corresponding to primitive *N*-linked glycans (Fig. 3), b) a series of hexose oligomers glucans with m/z ratios in agreement with degrees of polymerization ranging from 6 to 27 and hexose residues at the reducing end (glycogen, dextrin, and/or mannan type oligomers present as free oligosaccharides) and c) Two series of putative pentosan oligomers, which are consistent with the monosaccharide analysis showing a large amount of arabinose. In the *O*-linked analysis the mass spectrum was very complex (Figure S4) and a total of 80 *O*-linked glycans were detected. These glycans can be separated into 2 types: a) Mucin-type glycans featuring *N*-acetylhexosamine and hexose residues only (Fig. 3) and b) Pentose-containing glycans divided in 11 distinct series, each series showing a different reducing end. It is notable that the high-mannose type *N*-linked glycans and mucin type *O*-linked glycans detected in the mucus coming from *Pelagia noctiluca* are also typically detected in mammalian species. However, the glycan profile of this mucus showed high levels of pentose-containing oligomers, which are not typically found in mammalian systems but are consistent with high arabinose content (monosaccharide analysis).

## Discussion

Through the use of jellyfish and considering the growing use of nanomaterials and their associated risks, this study focused on the accumulation of nanoparticles by mucus substances secreted by several diploblastic metazoans e.g. cnidarians, including *Aurelia aurita*, *Pelagia noctiluca*, and Ctenophorans *Mnemiopsis leidyi.* Our results indicate that biomolecules produced by jellyfishes in certain circumstances interact favorably with NPs.

On the negative side, jellyfish blooms affect economies through effects on tourism^36^,^37^,^38^,^39^,^40^, clogging the coolant seawater intake of desalination (and power generation) plants^41^, interfere with coastal fish mariculture pens^37^, consume eggs and larvae of commercially important species^42^, and reducing fishing efforts^36^. These negative effects were mainly in Asia, though it has been assumed that those damages are more widespread than reported in the literature^36^. The enormous biomass of jellyfish can have a large impact on carbon, nitrogen and phosphorus global cycling^43^. However, their interaction with very small particles coming from natural and commercial sources remains poorly known^44^. Nanoparticles, such as metal oxide found in paint, cosmetics, animal feeds and fertilizers and, much like TiO_2_-NPs, which are commonly employed in sunscreens, can be dispersed in the environment. These NPs may enter sea water either directly through aerial deposition or indirectly e.g. *via* river systems. It has been reported that jellyfish often capture suspended food particles by swimming upwards, spreading their tentacles and oral arms and then sinking^45^. Some species produce nets of mucus to trap food particles^46^. Also, as reported by Hanaoka *et al.*, jellyfish (*A. aurita*) release blobs of mucus that capture suspended matter and sink down the water column^32^. This material can transport food and trace metals to the benthos.

Jellyfish are known for producing large amounts of mucus. This colloidal material originates from cells in the epidermis and gastrodermis^47^. However, less is known about its biochemical composition, which has been analyzed for few animals. In *A. aurita*, the analysis of the mucus revealed the presence of proteins, lipids, and carbohydrates similar to the mucus composition of other cnidarians^48^. Gelatinous metazoans also produce glycoproteins; for example *M. leidyi* releases modified aminosugar disaccharide metabolites^49^. More recently, Masuda *et al.* extracted mucus from five species of jellyfish and found it was rich in a family of sugary proteins called qniumucins^31^. In the case of the mucus secreted by *Pelagia noctiluca,* our analysis show high-mannose type *N*-linked glycans, mucin type *O*-linked glycans and high levels of pentose-containing oligomers.

We develop the concept for a potential role of mucus glycoproteins and/or glycans in the capture of very small particles, namely nanoparticles, and the decontamination of aqueous suspensions specifically containing nanowaste. The action of the glycoproteins, which constitute an important part of mucins is to capture the nanoparticles through interactions of electric charges. Glycoprotein macromolecules possess a variety of charges (thanks to positive *N*-bonds or negative O or P or S or C-O-bonds), which allow particles to link together, resulting in a Zero Point of Charge as pH ~2 for SiO_2_, pH ~5 for gold, pH ~6.5 for TiO_2_, pH ~8 for Fe_2_O_3_. Thus, the accumulation of nanoparticles in jellyfish mucus can be simply explained by the strong interactions occurring between NPs surfaces and glycoproteins and or glycans present in the mucus. The adsorption of nanoparticles on weak polyelectrolytes has been previously reported^50^,^51^. The charge distribution in the polymers, molecular weight^52^, and polymer conformation^53^ play an important role in the formation and density of the aggregates. Likewise, the chemical nature of the glycoproteins and/or glycans secreted by jellyfish allows the formation of strong interactions with NP surfaces. As reported recently for synthetic nanoparticles-polysaccharide interactions^54^, hydrogen bonding, ionic interactions, and dehydration of polar groups would be the key contributions to the strong affinity observed for the nanoparticle-biomolecules. One possible explanation of the unique trapping properties of the mucus would be a thermodynamically favorable evolution of the system. Indeed, the interactions of the nanoparticles with the 3D network of the mucus would lead to a more stable state corresponding to nanoparticles attached to the mucus.

## Conclusion

Several fundamental questions are emerging from the production of large amounts of nanoparticles linked to the industrial market. A global survey on the risk issues associated with NPs and their production remains to be completed, but it is clear that nanomaterials can potentially induce adverse effects on biological systems, including human and ecological spheres. As reported in this contribution jellyfish mucus can quantitatively trap nanoparticles; accumulations which would be inaccessible *via* filtration approaches. This attribute suggests that biomolecules belonging to the glycoproteins and/or glycan family could provide new opportunities to build decontamination systems for specific nano-waste treatments in factories using those nanotechnologies. By using the mucus secreted by jellyfish, the removal of nanoparticles below 50 nm in diameter from aqueous colloidal suspensions was successfully achieved at room temperature. Beside the potential environmental impact of the bioaccumulation of nanoparticles, this discovery opens up new practical avenues in removing nanoparticles from aqueous samples. Finally, our experiments provide a new way of viewing the fate of NP wastes arising from biomolecule-nanoparticle interactions. From our knowledge there is no filtration/flocculation system available on the market capable of removing quantitatively NPs from aqueous suspensions and this paper represents a first step in the rational design of efficient decontamination systems involving both natural and synthetic molecules.

## Methods

### Material

Three species of gelatinous zooplankton were collected alive from the bay of Villefranche-sur-Mer, France (43.696^o^N, 7.307^o^E) by hand-net and kayak during 2013 (see SI movie). All individuals were maintained in the laboratory at 18 °C in 15 L buckets of 1 μm filtered seawater, with the water changed daily. Each species produced mucus in a different way: the warty comb jelly *Mnemiopsis leidyi* (A. Agassiz, 1860), produced mucus as it died or when stressed; the mauve stinger *Pelagia noctiluca* (Forsskål, 1775) produced strings of mucus on a daily basis during reproduction^29^ and when stressed; the moon jellyfish *Aurelia aurita* (L., 1758) produced mucus as a stress response each time the jellyfish was handled or disturbed significantly (i.e. when transferred into clean water). All mucus was collected manually with a 4 mm diameter glass pipette (see “collection of the mucus”) within 1 hour of production and tested with Gold NP prior to storing in plastic bottles at −20 °C.

### Collection of the mucus

All type of mucus could be easily collected by gently stirring the water (in the bucket in which jellyfishes were maintained) with a glass pipette. The mucus then agglomerates on the pipette a little like when collecting cotton candy with a wood stick. It can then be concentrated and directed to the surface of the bucket and collected (pipetted in several times with the help of a little bucket because if not all the mucus is collected it would go back by himself in the mother tank-but see the video).

### Mucus production

(i) The *Aurelia aurita* shown in the video produced several hundreds of mL per day (if stressed). The quantity decreases while the jellyfish decrease in size. (ii) The *Pelagia noctiluca* “reproduction” mucus: A 8 cm female have a daily production of about 10–15 mL (depending on the age since collection). Reproduction occurs 3.5–4 hours after sunrise (or the start of illumination in the lab).

### Mucus incubated with NPs

The encapsulated quantum dots used for this study were synthesized according to the literature procedure^28^. In the case of QDs, jellyfish and encapsulated QDs solution in water (1 mL, concentration 17 μg/mL) were added to a 5 mL glass tube. The solution was softly shaken for couple of seconds and allowed to settle for 2–3 days. The mucus testing was routinely done using uncapped gold nanoparticles (concentration 10^−4^ M) and L-lysine capped gold nanoparticles (AuNPs) (concentration 10^−4^ M)^30^. To the solution of AuNPs, fresh mucus was added at a concentration of 2 parts AuNPs to 1 part mucus. This mix was mixed for 30–40 seconds (<1min) using a Vortex and the encapsulation of AuNPs by the mucus was assessed visually thanks to the pink-dark purple colour of the AuNPs.

### Osidic composition of mucus

Prior to analysis, jellyfish mucus were first centrifuged at 6,000 rpm for 10 min to separate the insoluble material from the insoluble one. Soluble fractions were subjected to ultrafiltration using a Millipore^®^ system with 1,000 Da cut-off membrane, washed with Milli-Q^®^ water, concentrated and finally lyophilized. Insoluble materials were simply lyophilized. Methanolysis was performed on both soluble and insoluble materials in 3 M MeOH–HCl at 100 °C for 4 h, and the resulting methylglycosides were N-acetylated and converted to the corresponding trimethylsilyl derivatives as described by Montreuil *et al.*^55^ (1986). GC analyses were performed with a 6850 GC System (Agilent Technologies™, USA) gas chromatograph with a HP-5MS cross-linked 5% Phenyl Methyl-polysiloxane (30 m, 0.25 mm i.d., 0.25 m film thickness) capillary column. The program used was 120 °C for 1 min, then a programmed temperature ramp to 200 °C (heating rate 3 °C/min to 180 °C, then 3 °C/min to 200 °C, held for 5min). The carrier gas was Nitrogen (1.0 ml/min) in the splitless mode.

### Oligosaccharide analyses

The Mucus samples (approx. 250 mL) were shaken in 0.2% NaCl aqueous solution (375 mL, 1.5 times (*v*/*v*), in Milli-Q^®^ water) at 4 °C for 48 h. Centrifugation was carried out to remove unwanted insoluble materials. After centrifugation, a gel-like precipitate was obtained upon the addition of three times the volume of EtOH (1875 mL) to the liquid. After standing overnight at 4 °C, the precipitates were separated by centrifugation and equally divided into four tubes with the help of Milli-Q^®^ water. Finally, lyophilization of the solution yielded crude samples. Four tubes, containing lyophilized extracts from jellyfish, were analyzed. After reconstituting each tube in 500 μL water per tube, some insoluble material still remained. The samples were therefore centrifuged to remove insoluble material prior to testing for protein concentration using a BCA as say. The estimated protein concentration of the supernatant was 0.58 mg/mL. The solution was concentrated to approximately 1.3 mg/mL and subjected to *N*- and *O*-glycan analysis.

## Additional Information

**How to cite this article**: Patwa, A. *et al.* Accumulation of nanoparticles in “jellyfish” mucus: a bio-inspired route to decontamination of nano-waste. *Sci. Rep.* **5**, 11387; doi: 10.1038/srep11387 (2015).

## Supplementary Material

Supplementary Information

Supplementary Movie S1

## Figures and Tables

**Figure 1:**
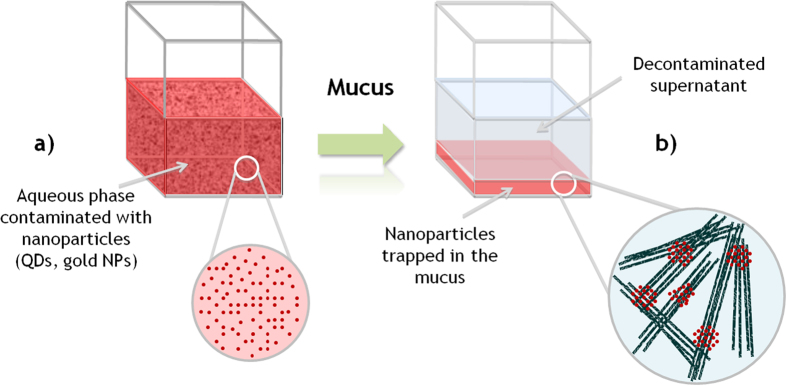
Schematic representation of the removal of NPs from an aqueous suspension using mucus secreted by jellyfish (*Pelagia noctiluca*). As reported in this contribution, mucus materials can capture quantitatively the NPs present in water (**a**). The NPs trapped by the mucus layer at the bottom of the sample releasing a decontaminated supernatant (**b**).
