# Supplementary material for: Accumulation of nanoparticles in “jellyfish” mucus: a bio-inspired route to decontamination of nano-waste
Source: Sci Rep. 2015 Jun 22;5:11387. doi: 10.1038/srep11387 (PMC4476112; doi:10.1038/srep11387)
Supplement: Supplementary Information [file srep11387-s1.pdf]

Accumulation of nanoparticles in "jellyfish" mucus: a bio-inspired route to decontamination of nano-waste

Amit Patwa[a,b], Alain Thiéry[c], Fabien Lombard[d], Martin K.S. Lilley[c,d], Claire Boisset[e], Jean-François Bramard[f], Jean-Yves Bottero[g] and Philippe Barthélémy*[, a,b]

[a]INSERM U869, Bordeaux, F-33076, France,
[b]Université de Bordeaux, Bordeaux, F-33076, France.
[c]IMBE UMR CNRS 7263, Aix-Marseille Université, Aix en Provence F-13545, France
[d]Sorbonne Universités, UPMC Univ Paris 06, UMR 7093, LOV, Observatoire océanologique, 06230, Villefranche sur mer, France
[e]CERMAV, CNRS UPR 5301, Grenoble, F-38041, France
[f]Cambulle, 171C, av. de la Mounine, 13320 Bouc Bel-Air
[g]CEREGE Europole de l'Arbois BP 80, Aix en Provence F-13545, France.

*__Corresponding author__:

Philippe Barthélémy

Telephone: 33 (0)5 57 57 48 53

Fax: 33 (0)5 57 57 10 15

Email: philippe.barthelemy@inserm.fr

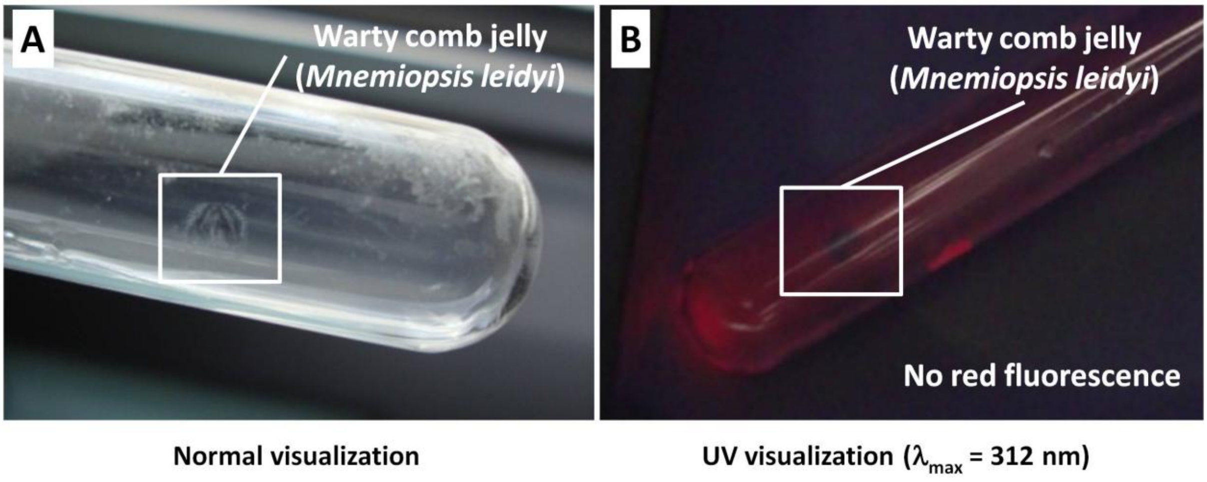

Figure S1: Warty comb jelly (*Mnemiopsis leidyi*) immediately after mixing with quantum dots (QDs); (A) normal visualization, (B) under UV visualization (λ_{max}=312 nm).

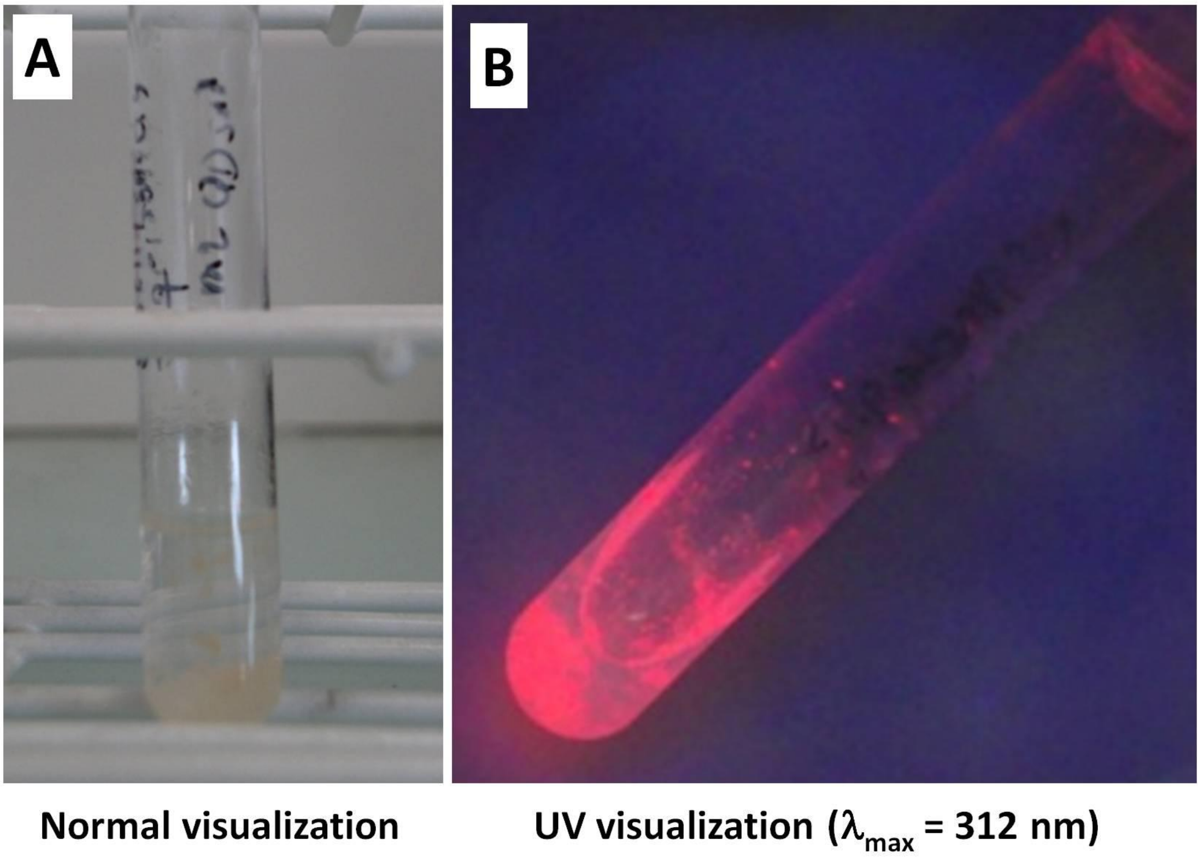

Figure S2: Jellyfish (*Mnemiopsis leidyi*) with quantum dots (QDs) after 72 h; (A) normal visualization, (B) under UV visualization (λ_{max}=312 nm).

N-Glycan profile of reproduction mucus:

The spectrum below shows the *N*-linked glycan profile of the jellyfish sample analyzed in this study. Selected peaks are annotated as follows: H – hexose oligomers, M – high-mannose type glycans, X – artifact of the assay, Istd – internal standard, A – pentosan series A, B - pentosan series B, and U – unknown. All spectra were collected on a MALDI-TOF mass spectrometer in reflectron mode, which yields high mass accuracy and isotopic resolution for more accurate glycan identification.

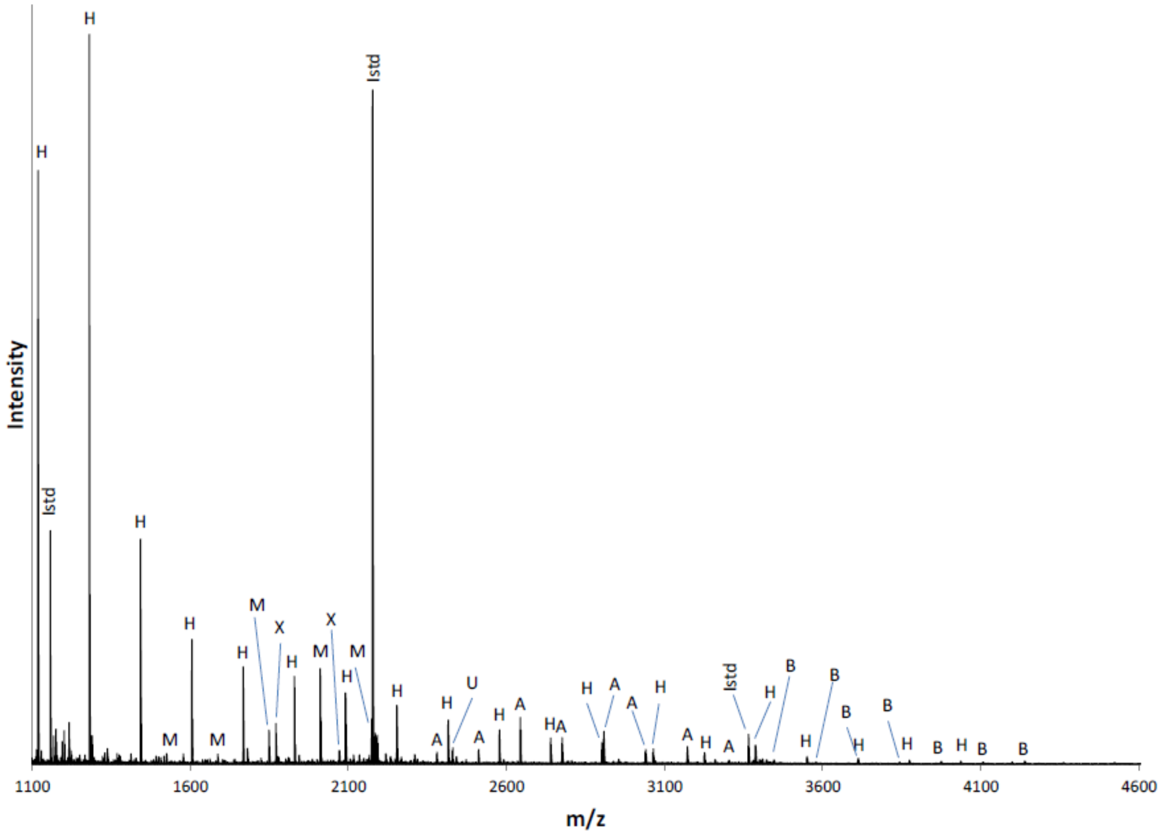

Figure S3: *N*-glycan profile of reproduction mucus

O-Glycan profile of reproduction mucus:

The spectrum below shows the *O*-linked glycan profile of the jellyfish sample analyzed in this study. Selected peaks are annotated with "Mu" (for Mucin type glycans) or the series label (A – K). Peaks labeled Bkg (background) were also detected in the *N*-linked glycan profile and are therefore probably *N*-linked glycans or free oligosaccharides. All spectra were collected on a MALDI-TOF mass spectrometer in reflectron mode, which yields high mass accuracy and isotopic resolution for more accurate glycan identification.

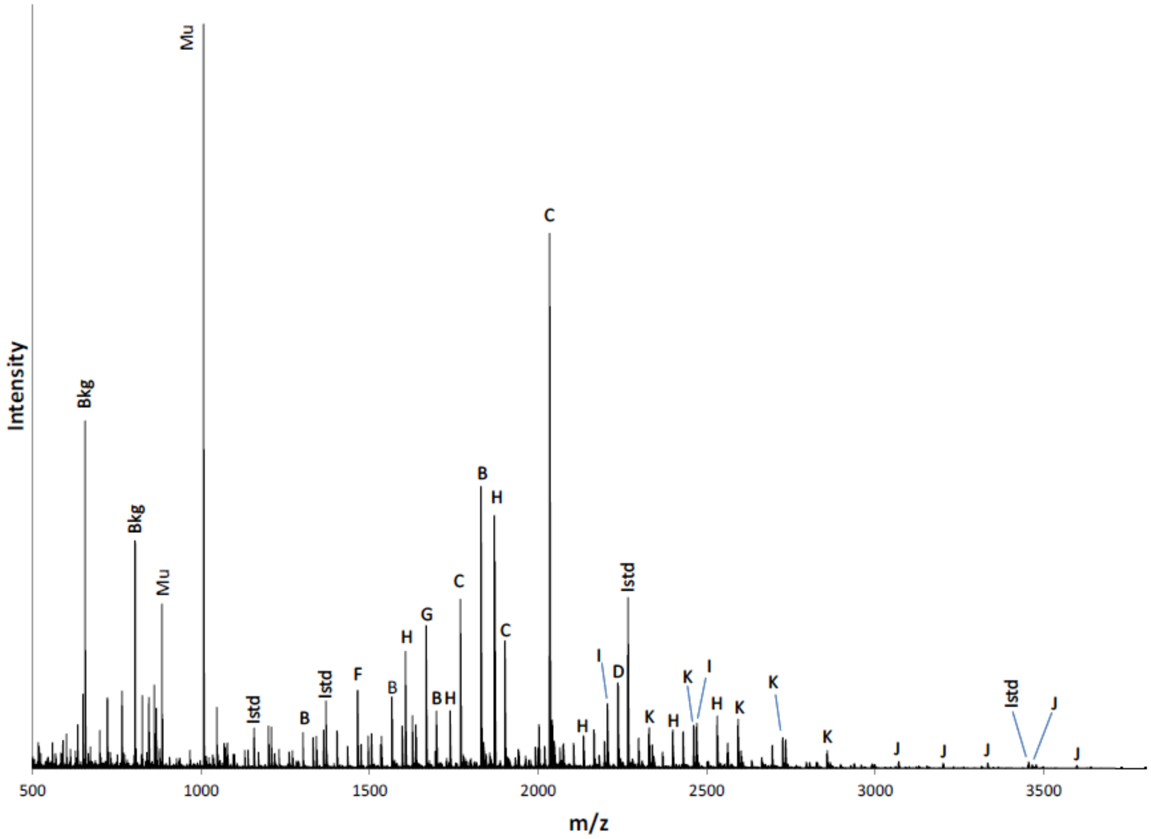

Figure S4: *O*-glycan profile of reproduction mucus

Symbol nomenclature of glycans:

Glycan structures are depicted as shown in Figure S5, using current convention. Glycan compositions are expressed as a five digit code, which represents the number of hexoses (Gal, Man, or Glc), *N*-acetylhexosamines (GlcNAc or GalNAc), deoxyhexoses (Fuc), *N*-acetylneuraminic acids (Neu5Ac), and *N*-glycolylneuraminic acids (Neu5Gc). Additional pentoses (arabinose, xylose, etc.) or modifications, such as sulfation, are indicated with a “+Pent” or “+SO_4”, respectively. The glycan shown in Figure S5 contains 5 hexoses, 4 *N*-acetylhexosamines, 1 fucose, 2 Neu5Ac, and 0 Neu5Gc and is therefore expressed as a glycan code of 5 4 1 2 0.

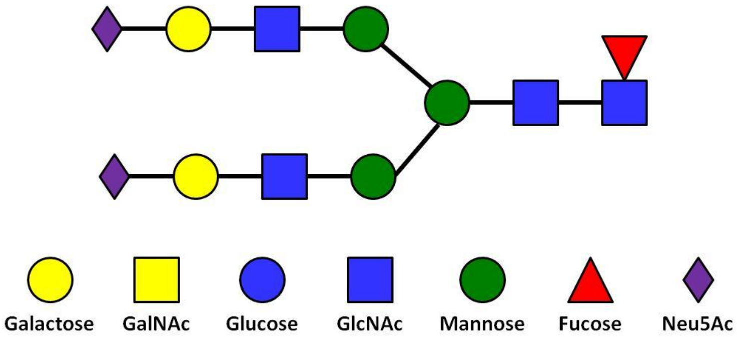

Figure S5: Symbol Nomenclature of the Consortium for Functional Glycomics

Movie Caption:

Movie S1: Production and collection of mucus from “jellyfish” and accumulation of gold nanoparticles in the mucus (see the movie (NanoDecon.mov)).

Table S1: Molecular Weights (m/z), Glycan Codes (Composition), Proposed Structures, and Relative Concentrations of *N*-linked Glycans

This table shows the observed molecular weight (m/z), glycan composition, and relative amount of each *N*-linked glycan detected in the jellyfish samples. Some peaks could not be positively identified based on mass and are noted either as part of a series or as an unknown.

m/z	Composition	Relative Amount	Proposed Structure(s)	
	(Hex HexNAc Fuc Neu5Ac Neu5Gc)	(% of total peak intensity)		
High-mannose Type Glycans				
1524.53	6 2 0 0 0	0.45	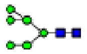	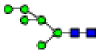
1686.59	7 2 0 0 0	0.38	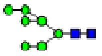	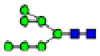
1848.64	8 2 0 0 0	1.54	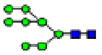	
2010.70	9 2 0 0 0	4.14	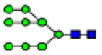	
2172.75	10 2 0 0 0	2.01	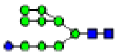	
Hexose Series				
1118.38	6 0 0 0 0	16.76		
1280.43	7 0 0 0 0[1]	22.05		
1442.48	8 0 0 0 0	7.50		
1604.53	9 0 0 0 0	4.62		
1766.59	10 0 0 0 0	4.08		
1928.64	11 0 0 0 0	3.95		
2090.69	12 0 0 0 0	3.60		
2252.74	13 0 0 0 0	3.10		
2414.80	14 0 0 0 0	2.34		
2576.85	15 0 0 0 0	1.83		
2738.90	16 0 0 0 0	1.43		
2900.96	17 0 0 0 0	1.09		
3063.01	18 0 0 0 0	0.82		
3225.06	19 0 0 0 0	0.68		
3387.11	20 0 0 0 0	1.64		
3549.17	21 0 0 0 0	0.36		
3711.22	22 0 0 0 0	0.51		
3873.27	23 0 0 0 0	0.23		
4035.33	24 0 0 0 0	0.22		
4197.47	25 0 0 0 0	0.20		
4359.43	26 0 0 0 0	0.16		
4521.67	27 0 0 0 0	0.11		

[1] This peak is a mixture of the endogenous hexose multimer and an internal standard that was spiked into the sample.

Table S1 continued…

m/z	Composition (Hex HexNAc Fuc Neu5Ac Neu5Gc)	Relative Amount (% of total peak intensity)	Proposed Structure(s)
Pentose Series A			
2246.82	pentosan series A	0.28	
2378.86	pentosan series A[2]	0.46	
2510.90	pentosan series A	0.81	
2642.91	pentosan series A	2.37	
2775.03	pentosan series A	1.41	
2907.00	pentosan series A	1.82	
3039.11	pentosan series A	0.94	
3171.13	pentosan series A	0.93	
3303.17	pentosan series A	0.32	
Pentose Series B			
3445.27	pentosan series B	0.34	
3577.29	pentosan series B	0.18	
3709.33	pentosan series B	0.19	
3841.42	pentosan series B	0.14	
3973.41	pentosan series B	0.21	
4105.49	pentosan series B	0.17	
4237.50	pentosan series B	0.20	
Unknown Peaks			
1491.38	unknown	0.34	
1577.55	unknown	0.39	
1780.64	unknown	0.48	
1943.66	unknown	0.40	
2164.76	unknown	0.74	
2428.84	unknown	0.68	
2440.85	unknown	0.38	

[2] This peak could also be A2 (glycan 54020) but, since no sialic acid has been detected in these samples, is it more likely part of the pentose series.

Table S2: Molecular Weights (*m/z*), Glycan Codes (Composition), Proposed Structures, and Relative Concentrations of *O*-linked Glycans

This table shows the observed molecular weight (*m/z*), glycan composition, and relative amount of each *O*-linked glycan detected in the jellyfish samples. Some peaks could not be positively identified based on mass and are noted either as part of a series or as an unknown.

m/z	Composition	Relative Amount	Proposed Structure(s)	
	(Hex HexNAc Fuc Neu5Ac Neu5Gc)	(% of total peak intensity)		
Mucin-type O-linked Glycans				
601.14	1 1 0 0 0	0.47	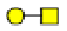	
804.22	1 2 0 0 0	2.97	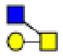	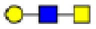
1007.30	1 3 0 0 0	9.72	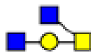	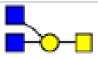
O-Linked Series A Glycans				
967.26	0 1 0 0 0 + Pent$_4$	0.37		
1099.31	0 1 0 0 0 + Pent$_5$	0.25		
1231.35	0 1 0 0 0 + Pent$_6$	0.34		
1363.39	0 1 0 0 0 + Pent$_7$	0.72		
1495.43	0 1 0 0 0 + Pent$_8$	0.62		
1627.47	0 1 0 0 0 + Pent$_9$	1.14		
O-Linked Series B Glycans				
906.25	0 2 0 0 0 + Pent$_2$	0.20		
1170.34	0 2 0 0 0 + Pent$_4$	0.27		
1302.37	0 2 0 0 0 + Pent$_5$	0.68		
1434.42	0 2 0 0 0 + Pent$_6$	0.43		
1566.47	0 2 0 0 0 + Pent$_7$	1.40		
1698.53	0 2 0 0 0 + Pent$_8$	1.22		
1830.56	0 2 0 0 0 + Pent$_9$	6.25		
O-Linked Series C Glycans				
1505.45	0 3 0 0 0 + Pent$_5$	0.69		
1637.51	0 3 0 0 0 + Pent$_6$	0.92		
1769.58	0 3 0 0 0 + Pent$_7$	3.60		
1901.61	0 3 0 0 0 + Pent$_8$	2.98		
2033.66	0 3 0 0 0 + Pent$_9$	12.67		
2165.69	0 3 0 0 0 + Pent$_{10}$	1.17		
2297.73	0 3 0 0 0 + Pent$_{11}$	0.97		
2429.77	0 3 0 0 0 + Pent$_{12}$	1.26		
2561.80	0 3 0 0 0 + Pent$_{13}$	0.89		
2693.87	0 3 0 0 0 + Pent$_{14}$	0.79		
2825.90	0 3 0 0 0 + Pent$_{15}$	0.26		

Table S2 continued…

m/z	Composition	Relative Amount	Proposed Structure(s)
	(Hex HexNAc Fuc Neu5Ac Neu5Gc)	(% of total peak intensity)	
O-Linked Series D Glycans			
2104.68	0 4 0 0 0 + $Pent_8$	0.71	
2236.72	0 4 0 0 0 + $Pent_9$	2.44	
2368.73	0 4 0 0 0 + $Pent_{10}$	0.49	
O-Linked Series E Glycans			
1129.30	1 1 0 0 0 + $Pent_4$	0.33	
1261.34	1 1 0 0 0 + $Pent_5$	0.33	
O-Linked Series F Glycans			
936.26	1 2 0 0 0 + $Pent_1$	0.18	
1068.31	1 2 0 0 0 + $Pent_2$	0.43	
1200.34	1 2 0 0 0 + $Pent_3$	0.74	
1332.38	1 2 0 0 0 + $Pent_4$	0.56	
1464.43	1 2 0 0 0 + $Pent_5$	1.51	
1596.48	1 2 0 0 0 + $Pent_6$	0.90	
O-Linked Series G Glycans			
1139.35	1 3 0 0 0 + $Pent_1$	0.38	
1271.39	1 3 0 0 0 + $Pent_2$	0.34	
1403.41	1 3 0 0 0 + $Pent_3$	0.71	
1535.46	1 3 0 0 0 + $Pent_4$	0.64	
1667.52	1 3 0 0 0 + $Pent_5$	2.81	
O-Linked Series H Glycans			
1342.42	1 4 0 0 0 + $Pent_1$	0.58	
1474.47	1 4 0 0 0 + $Pent_2$	0.49	
1606.52	1 4 0 0 0 + $Pent_3$	2.17	
1738.55	1 4 0 0 0 + $Pent_4$	1.24	
1870.60	1 4 0 0 0 + $Pent_5$	5.45	
2002.64	1 4 0 0 0 + $Pent_6$	1.19	
2134.71	1 4 0 0 0 + $Pent_7$	1.01	
2398.77	1 4 0 0 0 + $Pent_9$	1.17	
2530.81	1 4 0 0 0 + $Pent_{10}$	1.51	
2662.87	1 4 0 0 0 + $Pent_{11}$	0.32	

Table S2 continued…

m/z	Composition	Relative Amount	Proposed Structure(s)
	(Hex HexNAc Fuc Neu5Ac Neu5Gc)	(% of total peak intensity)	
O-Linked Series I Glycans			
1941.63	1 5 0 0 0 + $Pent_4$	0.47	
2073.69	1 5 0 0 0 + $Pent_5$	0.66	
2205.73	1 5 0 0 0 + $Pent_6$	1.79	
2337.77	1 5 0 0 0 + $Pent_7$	0.76	
2469.81	1 5 0 0 0 + $Pent_8$	1.39	
2601.84	1 5 0 0 0 + $Pent_9$	0.64	
2733.90	1 5 0 0 0 + $Pent_{10}$	1.02	
O-Linked Series J Glycans			
2804.96	1 6 0 0 0 + $Pent_9$	0.21	
2936.98	1 6 0 0 0 + $Pent_{10}$	0.18	
3069.02	1 6 0 0 0 + $Pent_{11}$	0.22	
3201.08	1 6 0 0 0 + $Pent_{12}$	0.17	
3333.10	1 6 0 0 0 + $Pent_{13}$	0.18	
3465.17	1 6 0 0 0 + $Pent_{14}$	0.13	
3597.22	1 6 0 0 0 + $Pent_{15}$	0.13	
O-Linked Series K Glycans [1]			
2064.65	0 2 0 0 0 + $Pent_{10}$ + SO_4	0.57	
2196.69	0 2 0 0 0 + $Pent_{11}$ + SO_4	0.88	
2328.74	0 2 0 0 0 + $Pent_{12}$ + SO_4	1.29	
2460.77	0 2 0 0 0 + $Pent_{13}$ + SO_4	1.40	
2592.88	0 2 0 0 0 + $Pent_{14}$ + SO_4	1.58	
2724.87	0 2 0 0 0 + $Pent_{15}$ + SO_4	1.09	
2856.91	0 2 0 0 0 + $Pent_{16}$ + SO_4	0.60	
Unknown Peaks			
333.98	unknown	0.27	
672.03	unknown	0.29	
765.17	unknown	1.10	
876.20	unknown	0.27	
1218.28	unknown	0.28	
1999.64	unknown	0.56	

[1] While the molecular weight of these series is consistent with the presence of a sulfate (SO_4) residue, other modifications are also possible.

Monosaccharide analysis

The soluble and insoluble fractions of *Pelagia noctiluca* (reproduction mucus) were analyzed. No sugar has been identified in the soluble fraction. Results of the glycoside composition of the insoluble fraction obtained by gas chromatography after methanolysis are presented in the tables below: the saccharide composition is expressed as mass percentage (Table S3) and molar ratio (Table S4). Assays were performed in triplicate. The mucus is mainly constituted of arabinose, mannose, glucose, *N*-acetyl glucosamine and *N*-acetyl galactosamine.

Table S3 : Composition (dried mass percent) of polysaccharides contained in the insoluble mucus fraction of *Pelagia noctiluca*. The glycosyl residue composition was determined by gas-liquid chromatography analysis of trimethylsilyl derivatives after methanolysis. Results are expressed in percentage in mass. Ara : arabinose; Glc : glucose; Man : mannose; GalNAc : *N*-acetyl galactosamine; GlcNAc : *N*-acetyl glucosamine.

Monosaccharide / Sample	Ara	Glc	Man	GalNAc	GlcNAc	% hydrolysis
Insoluble mucus	1.7 ± 0.1	0.6 ± 0.1	1 ± 0.15	2 ± 0.4	1.8 ± 0.35	7.1 ± 0.7

Table S4 : Composition (molar ratio) of polysaccharides contained in the insoluble mucus fraction of *Pelagia noctiluca*. The glycosyl residue composition was determined by gas-liquid chromatography analysis of trimethylsilyl derivatives after methanolysis. Results are expressed in percentage in mass.The results are presented in the form of ratios, using arabinose, the most abundant monosaccharide present in insoluble mucus, as the standard. Ara : arabinose; Glc : glucose; Man : mannose; GalNAc : *N*-acetyl galactosamine; GlcNAc : *N*-acetyl glucosamine.

Monosaccharide / Sample	Ara	Glc	Man	GalNAc	GlcNAc
Insoluble mucus	1	0.3 ± 0.03	0.5 ± 0.07	0.8 ± 0.15	0.7 ± 0.2

Gas-liquid chromatogram

The sample was heated 4 hrs at 100°C in 3N methanolic HCl, and the products were converted to TMS derivatives. The silylated monosaccharides were separated on a HP-5MS colunn, with nitrogen as gas vector. Myo-inositol was used as internal standard.

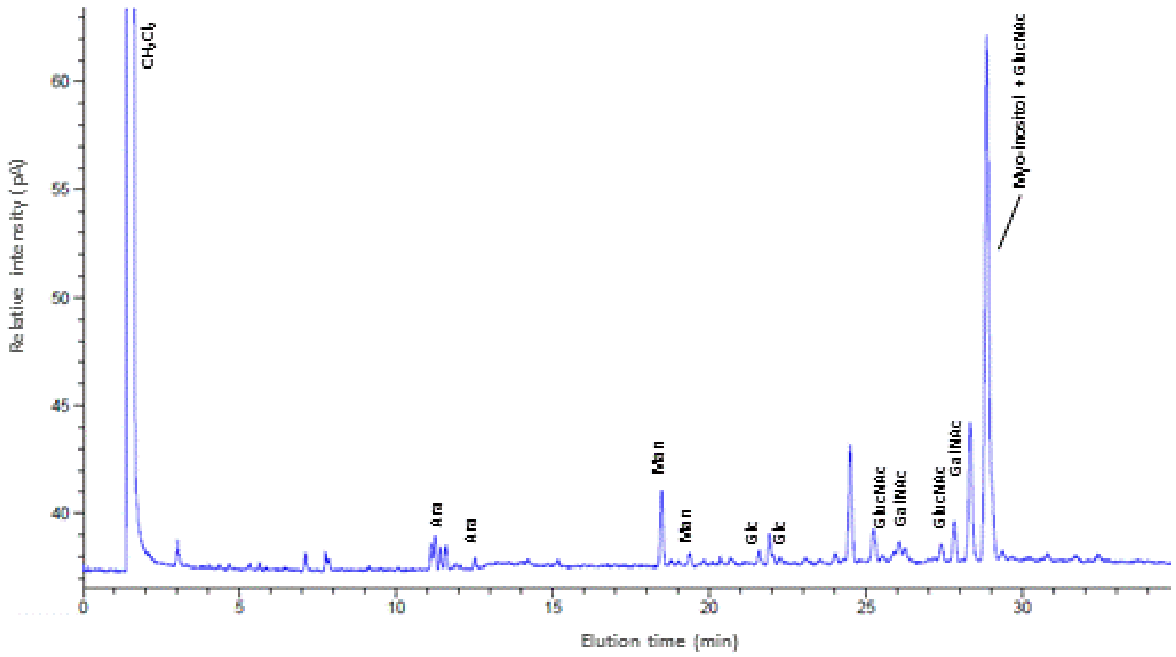

Figure S6: Gas-liquid chromatogram of the insoluble mucus fraction of *Pelagia noctiluca*. Ara : arabinose; Man : mannose, Glc : glucose; GlcNAc : *N*-acetyl glucosamine; GalNAc : *N*-acetyl galactosamine
